# Colonic luminal microbiota and bacterial metabolite composition in pregnant Huanjiang mini-pigs: effects of food composition at different times of pregnancy

**DOI:** 10.1038/srep37224

**Published:** 2016-12-05

**Authors:** Xiang-feng Kong, Yu-jiao Ji, Hua-wei Li, Qian Zhu, F. Blachier, Mei-mei Geng, Wen Chen, Yu-long Yin

**Affiliations:** 1 Key Laboratory of Agro-ecological Processes in Subtropical Region, Hunan Provincial Engineering Research Center for Healthy Livestock, Institute of Subtropical Agriculture, Chinese Academy of Sciences, Hunan 410125, China; 2 Research Center of Mini-pig, Huanjiang Observation and Research Station for Karst Ecosysterm, Chinese Academy of Sciences, Huanjiang, Guangxi 547100, China; 3 INRA, CNRH-IdF, AgroParisTech, UMR 914 Nutrition Physiology and Ingestive Behavior, Paris 75005, France

## Abstract

The gut harbours diverse and complex microbiota, which influence body health including nutrient metabolism, immune development, and protection from pathogens. Pregnancy is associated with immune and metabolic changes that might be related to microbiota compositional dynamics. We therefore investigated the colonic luminal bacteria community in Huanjiang mini-pigs fed diets with different nutrient levels from the first to third trimester of pregnancy. The concentrations of intestinal metabolites including short-chain fat acids, NH_3_-N, indole, skatole, and bioamines were also determined. We found that the colonic bacteria species richness estimators (Chao1 and ACE) decreased with increased gestational age. The dominant phyla identified were Firmicutes and Bacteroidetes; the dominant genera were *Lactobacillus, Treponema, Ruminococcus, Clostridium*, and *Prevotella*. In addition, microbiota displayed spatial and temporal heterogeneity in composition, diversity, and species abundance in different colonic segments from the first to third trimester of pregnancy. Furthermore, the bacterial metabolites also changed according to the diet used and the pregnancy stage. These findings suggest that colonic bacteria richness decreased as gestational age increased, and that the higher nutrient level diet increased the production of metabolites related to nitrogen metabolism. However, although the higher nutrient diet was associated with pregnancy syndrome, causal links remain to be determined.

Dietary nutrients during gestation play important roles in the reproductive performance of sows and within-litter variation of their offspring^1^. It was initially thought that sows in early gestation should receive large nutrient amounts^2^. However, high nutrient levels during gestation can increase sow fat mass, resulting in farrowing problems, poor dietary intake during lactation, and less reproductive performance in the next cycle^3^. Notably, sows fed diets with high energy content during early gestation suffer embryo deaths^4^. Conversely, after mating, nutrition restriction supports progesterone rise and embryonic survival in gilts. Thus, excess or inadequate dietary energy and protein ingestion by pregnant pigs can contribute to poor reproductive performance and impaired physiological functions.

Uterine growth during gestation causes profound gastrointestinal tract modification, giving rise to altered gastric motility and intestinal transit time along with gastrointestinal disturbances^5^. Recently, Koren *et al*. demonstrated that in humans, the gut microbiota composition during first trimester pregnancy is very similar to that of a healthy non-pregnant control group but becomes enriched in Proteobacteria and Actinobacteria during the third trimester^6^. Notably, these latter changes are similar to those detected in inflammatory bowel disease and obesity. In the gut, the bacteria load also increases over the course of gestation^7^. However, Jost *et al*. reported little or no modifications of the faecal microbiota composition during early and late pregnancy^8^. In humans, disorders associated with gut microbiota dysbiosis include obesity^9^, type 2 diabetes^10^, and malnutrition^11^. The mammalian gut, especially the hindgut, is colonised by a large number of microbes with varying microbial composition in the different parts of the gut^12^. The colon is colonised by up to almost 10^12^ bacteria per gram digesta^13^. The microbiota in the gut lumen plays an important role in the digestion and absorption of nutrients, prevention of pathogen colonisation, and maintenance and regulation of normal mucosal immunity^14^. Recently, the roles of gut microbiome activity and their metabolites in regulating host physiological functions in association with longevity^15^ and the metabolic changes during pregnancy^6^ have attracted considerable interest. Intestinal microbiota composition is affected by multiple factors including environmental parameters, dietary composition in terms of quality and quantity, and antibiotics use^16^. Many dietary components including protein and amino acid-derived bacterial metabolites such as short-chain fatty acids, NH_3_-N, indole, skatole, and bioamines affect the relationship between gut microbiota and the intestinal mucosa, and this relationship might evolve according to life stage^17^.

In the present study, we utilised the working hypothesis that sows fed a diet with high nutrient level during the entire gestation period might experience microflora composition and metabolic activity changes in the colon, with possible dysfunctions such as signs of metabolic syndrome. Accordingly, we aimed to document the changing diversity and structure of the colonic lumimal bacteria community from the first to third trimester of gestation in Huanjiang mini-pigs fed relatively higher or lower nutrient level diets formulated to meet the nutrient requirements recommended by the National Research Council (NRC diet) or the Chinese National Feeding Standard for Swine (CNF diet), respectively. Additionally, colonic bacterial metabolites including short-chain fatty acids (SCFAs), NH_3_-N, indole, skatole, and bioamines were determined.

## Results

### Diversity of colon bacterial communities

The colon contents were sampled from 32 sows fed the NRC or CNF diets belonging to the three pregnancy stage groups. Paired-end reads generated from the original DNA fragments using Illumina MiSeq next generation sequencing were merged using FLASH, producing 1,328,640 and 1,323,342 row tags from the 32 proximal or distal colon content samples, respectively. After filtering for quality and removing apparent chimeras, 1,291,145 and 1,282,402 effective tags were acquired for analysis, averaging 40,348 and 40,075 sequences in every proximal and distal colon content sample, respectively. The average read length was 253 bp. Operational taxonomic units (OTUs) were defined as a bin of sequence reads sharing ≥97% nucleotide sequence identity. From the effective sequences, we detected 35,540 and 37,358 OTUs averaging 1,110.63 ± 123.57 and 1,167.44 ± 93.52 OTUs in every proximal and distal colon content sample, respectively.

The individual samples were normalised and the OTU table within each sample was rarefied to 31,465 sequence reads based upon the sample rarefaction curves. The normalised sequence reads were then used to calculate the species diversity (Shannon and Simpson indices), richness estimators (Chao1 and ACE), and the coverage (Good’s coverage estimator; Table 1). In the proximal colon content samples, the OTUs decreased significantly according to pregnancy stage and were affected by diet × stage conditions (*P* < 0.05). Chao1, ACE, and Coverage were decreased (*P* < 0.05) with later pregnancy stages, but the Simpson and Shannon indices were not affected by diet or stage. In the distal colon content samples, the OTUs displayed a decreasing trend (0.05 ≤ *P* < 0.10) and Chao1 and ACE were significantly decreased (*P* < 0.05) with pregnancy stage. Bacterial diversity showed no difference between colonic content samples (*P* > 0.05).

### Changes in the bacterial phylum, genera, and OTUs of colonic content microbiota

Principal coordinate analyses (PCoA) were conducted to determine the phylogenetic relationship between the tested microbial community samples (Supplementary Fig. S1). Using the detected OTUs, we determined whether the data separated into distinct sample clusters. The CNF and NRC diet groups could almost be separated using the proximal colon contents according to pregnancy stage. Pregnancy day 45, 75, and 110 proximal colon content samples were clearly separated in the NRC but not in the CNF diet group. No separation was detected in clusters using the distal colon contents (Supplementary Fig. S1).

**Figure 1:**
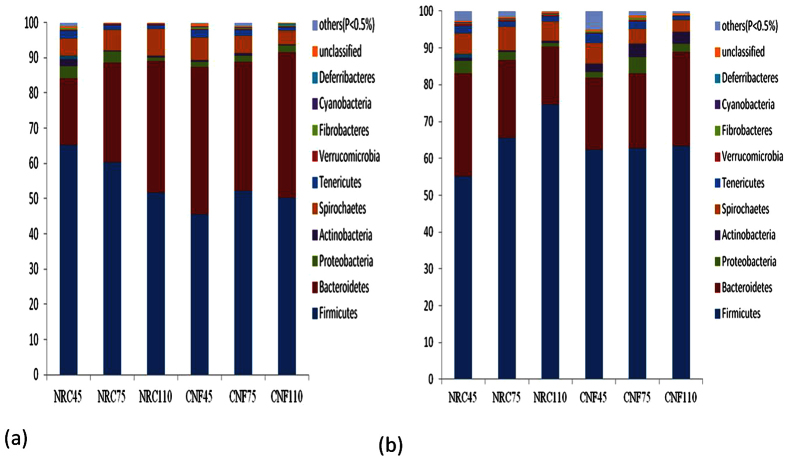
Taxonomic composition of the colon bacterial communities on the phylum level in Huanjiang mini-pigs at different stages of pregnancy (%). (**a**) Luminal content samples of the proximal and (**b**) distal colon. The taxonomic composition of the colon microbiota among the samples was compared based on the relative abundance (reads of a taxon/total reads in a sample). NRC45, NRC75, and NRC110: data from samples obtained from Huanjiang mini-pigs fed with the NRC diet for 45, 75, and 110 days, respectively. CNF45, CNF75, and CNF110: data from samples obtained from Huanjiang mini-pigs fed with the CNF diet for 45, 75, and 110 days, respectively.

At the phylum level, the abundance of 10 phyla in any sample was ≥0.5%; i.e., Firmicutes, Bacteroidetes, Proteobacteria, Actinobacteria, Spirochaetes, Tenericutes, Verrucomicrobia, Fibrobacteres, Cyanobacteria, and Deferribacteres. In the colonic samples, Firmicutes and Bacteroidetes were the two dominant phyla. Firmicutes were decreased in the proximal colon content samples but Bacteroidetes increased from pregnancy days 45 to 110 of sows fed the NRC diet. In comparison, the relative abundance of Firmicutes was higher, but Bacteroidetes was lower in NRC diet-fed than in CNF diet-fed sows (Fig. 1a). In distal colon content samples, the Firmicutes abundance increased whereas Bacteroidetes from pregnancy days 45 to 110 with the NRC diet decreased. The Firmicutes abundance was almost stable from pregnancy days 45 to 110 (Fig. 1b). Overall, the Firmicutes abundance was greater, but Bacteroidetes abundance was lower in the distal compared to the proximal colon content samples.

**Figure 2:**
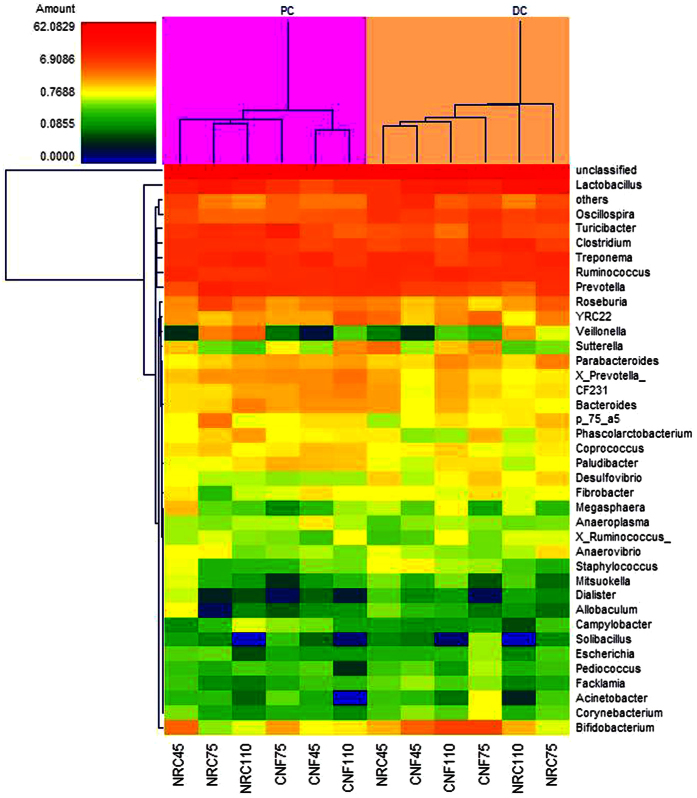
Genus-level taxonomic composition of the bacterial communities of colonic contents in Huanjiang mini-pigs at different pregnant stages (%). NRC45, NRC75, and NRC110: data from samples obtained from Huanjiang mini-pigs fed with the NRC diet for 45, 75, and 110 days, respectively. CNF45, CNF75, and CNF110: data from samples obtained from Huanjiang mini-pigs fed with the CNF diet for 45, 75, and 110 days, respectively.

The relative abundance of genera, among the 37 observed, was ≥0.5% in every sample (Fig. 2). In the proximal colon contents, within the Bacteroidetes phylum, the genus *Paludibacter* and *Parabacteroides* abundances were higher (*P* < 0.05) in the CNF vs. the NRC diet group. Within the Firmicutes phylum, the genera *Anaerovibrio, Lactobacillus, Mitsuokella*, and *Roseburia* abundances were lower (*P* < 0.05) in the CNF diet group. The unclassified genera changed significantly with diet (*P* < 0.05), with higher abundance in the CNF diet group. With increasing pregnancy time, *Corynebacterium, Allobaculum, Mitsuokella, Roseburia, Solibacillus, Staphylococcus, Acinetobacter*, and the unclassified genus changed significantly (*P* < 0.05). Within the Actinobacteria and Proteobacteria phyla, genera *Corynebacterium* and *Acinetobacter* abundance, respectively, decreased linearly with pregnancy stage (*P* < 0.05) as did genus *Allobaculum* abundance within the Firmicutes phylum. Unclassified genus and Firmicutes phylum *Mitsuokella* abundance peaked on pregnancy day 45 and exhibited its lowest level on pregnancy day 75 (*P* < 0.05), when Firmicutes *Roseburia* and *Solibacillus* abundances were highest (*P* < 0.05) but *Staphylococcus* was lowest (*P* < 0.05). Diet type interacted with the pregnancy stage for the genera of *Bifidobacterium, Allobaculum, Megasphaera, Mitsuokella, Roseburia, Solibacillus*, and *Staphylococcus*.

In the distal colon contents, within the Actinobacteria phylum, the genus *Bifidobacterium* abundance was higher (*P* < 0.05) in CNF vs. NRC diet groups. Within the Firmicutes phylum, the genera *Anaerovibrio, Dialister, Roseburia*, and *Veillonella* abundances were lower (*P* < 0.05) in the CNF diet groups. The effects of pregnancy stage on bacterial composition were as follows: within the Firmicutes phylum, the genera *Allobaculum, Dialister, Lactobacillus, Megasphaera, Mitsuokella, Staphylococcus*, and [*Ruminococcus*] abundances changed significantly with pregnancy stage, being lowest (*P* < 0.05) on pregnancy day 75 for *Allobaculum, Megasphaera*, and *Mitsuokella* and on pregnancy day 45 for *Dialister (P* < 0.05). *Lactobacillus* and [*Ruminococcus*] abundances increased whereas *Staphylococcus* abundance decreased linearly with pregnancy stage (*P* < 0.05). The unclassified genus abundance was lowest on pregnancy day 75 (*P* < 0.05) (Fig. 2).

We identified 4,010 OTUs in the colonic contents across the two dietary groups and three pregnancy stages. Consideration was only given to OTUs detected at abundances ≥0.5% in any sample. Overall, we identified 37 OTUs that differed in abundance in the proximal colon samples (Table 2), of which 18 showed differences with diet. Specifically, the abundances of OTU-2 (genus: *Lactobacillus*), OTU-9 (family: *Lachnospiraceae*), OTU-36 (g: *Treponema*), OTU-41 (g: *Roseburia*), OTU-45 (species: *Lactobacillus delbrueckii*), OTU-59 (g: *Treponema*), and OTU-79 (g: *Anaerovibrio*) were higher (*P* < 0.05) in NRC vs. CNF diet groups. Conversely, the abundances of OTU-27 (f: *Ruminococcaceae*), OTU-38 (order: *Clostridiales*), OTU-46 (g: *YRC22*), OTU-68 (f: *Lachnospiraceae*), OTU-82 (g: *Parabacteroides*), OTU-98 (g: *Oscillospira*), OTU-105 (o: *Bacteroidales*), OTU-106 (g: *Fibrobacter*), OTU-195 (g: *[Prevotella]*), OTU-1149 (p: *Bacteroidetes*), and OTU-2187 (f: *Sphingomonadaceae*) were lower (*P* < 0.05) in the NRC diet groups. Additionally, 18 OTUs demonstrated pregnancy stage-associated differences: OTU-17 (f: *Lachnospiraceae*), OTU-20 (g: *Oscillospira*), OTU-62 (g: *Prevotella*), OTU-65 (g: *Ruminococcus*), and OTU-1366 (f: *Christensenellaceae*) abundances linearly decreased (*P* < 0.05) as pregnancy processed whereas OTU-36 (g: *Treponema*), OTU-41 (g: *Roseburia*), OTU-46 (g: *YRC22*), OTU-55 (g: *Prevotella*), OTU-1149 (p: *Bacteroidetes*), and OTU-3683 (f: *Ruminococcaceae*) linearly increased (*P* < 0.05). Furthermore, the abundances of OTU-25 (f: *Ruminococcaceae*), OTU-28 (f: *Streptococcaceae*), OTU-33 (g: *Ruminococcus*), OTU-47 (f: *Lachnospiraceae*), OTU-85 (f: *Lachnospiraceae*), and OTU-216 (f: *S24-7*) were the lowest whereas OTU-68 (f: *Lachnospiraceae*) was the highest on pregnancy day 75 (*P* < 0.05).

Furthermore, we identified 45 OTUs with differing abundance in the distal colon samples (Table 3), of which 27 exhibited differences according to diet. The abundances of OTU-9 (f: *Lachnospiraceae*), OTU-14 (s: *Clostridium butyricum*), OTU-36 (g: *Treponema*), OTU-75 (f: *Lachnospiraceae*), OTU-133 (g: *Oscillospira*), OTU-1967 (g: *Prevotella*), and OTU-2531 (f: *Ruminococcaceae*) were higher (*P* < 0.05) in the NRC vs. CNF diet groups. Conversely, OTU-11 (g: *Bifidobacterium*), OTU-25 (f: *Ruminococcaceae*), OTU-28 (f: *Streptococcaceae*), OTU-34 (g: *Prevotella*), OTU-38 (o: *Clostridiales*), OTU-41 (g: *Roseburia*), OTU-49 (g: *Veillonella*), OTU-72 (f: *Ruminococcaceae*), OTU-77 (s: *Ruminococcus flavefaciens*), OTU-79 (g: *Anaerovibrio*), OTU-85 (f: *Lachnospiraceae*), OTU-113 (f: *Ruminococcaceae*), OTU-120 (class: *Clostridia*), OTU-164 (g: *Staphylococcus*), OTU-659 (g: *Oscillospira*), OTU-1428 (f: *Lachnospiraceae*), OTU-1890 (g: *Roseburia*), OTU-2133 (o: *Clostridiales*), OTU-2768 (o: *Clostridiales*), and OTU-3219 (f: *Christensenellaceae*) abundances were lower (*P* < 0.05) in the NRC diet groups. Of the 17 OTUs showing pregnancy stage differences, OTU-27 (f: *Ruminococcaceae*), OTU-48 (f: *Christensenellaceae*), OTU-1428 (f: *Lachnospiraceae*), OTU-1454 (g: *Ruminococcus*), OTU-2768 (o: *Clostridiales*), and OTU-3683 (f: *Ruminococcaceae*) abundances linearly increased (*P* < 0.05) with pregnancy progression. However, OTU-31 (s: *Lactobacillus reuteri*), OTU-62 (g: *Prevotella*), OTU-89 (g: *Prevotella*), OTU-1511 (s: *Lactobacillus reuteri*), OTU-1967 (g: *Prevotella*), and OTU-2590 (g: *Treponema*) abundances linearly decreased (*P* < 0.05). Additionally, OTU-35 (f: *[Paraprevotellaceae]*), OTU-146 (g: *Staphylococcus*), OTU-1366 (f: *Christensenellaceae*), and OTU-1386 (f: *Ruminococcaceae*) exhibited the highest and OTU-29 (s: *Megasphaera elsdenii*) the lowest abundances on pregnancy day 75 (*P* < 0.05).

### Diet, pregnancy stage, and colonic segment correlations with gut microbiota

At the phylum level, Firmicutes (*P* < 0.05) and Bacteroidetes (*P* < 0.05) in the proximal colon contents correlated with diet (Supplementary Table S1). Firmicutes was higher in the NRC than in the CNF diet group, whereas Bacteroidetes was the converse. Actinobacteria (*P* < 0.05) and Elusimicrobia (*P* < 0.05) in the distal colon contents correlated with diet. Actinobacteria was lower in the NRC diet group, and Elusimicrobia presented the opposite. Consistent with pregnancy stage and gut microbiota correlation, Verrucomicrobia (*P* < 0.05) and Tenericutes (*P* < 0.01) decreased with pregnancy stage in the proximal colon contents whereas in the distal colon contents Firmicutes (*P* < 0.05) increased but Tenericutes (*P* < 0.05) decreased. Firmicutes (*P* < 0.01), Actinobacteria (*P* < 0.05), Verrucomicrobia (*P* < 0.01), and Lentisphaerae (*P* < 0.05) were all elevated in the distal vs. the proximal colon contents, whereas Bacteroidetes (*P* < 0.01) presented the inverse.

At the genus level (Supplementary Table S2), consistent with the correlation between dietary characteristics and gut microbiota composition in the proximal colon contents, *Paludibacter* and *Parabacteroides* (p: Bacteroidetes) were lower (*P* < 0.05) in the NRC vs. the CNF diet group, whereas *Lactobacillus (P* < 0.01) and *Roseburia (P* < 0.05) (p: Firmicutes) as well as *Escherichia* (p: Proteobacteria) (*P* < 0.05) and *Anaeroplasma* (p: Tenericutes) (*P* < 0.05) were higher in the NRC. In the distal colon contents, *Bifidobacterium* and *Corynebacterium* (p: Actinobacteria) were lower (*P* < 0.05) in the NRC vs. the CNF diet group; *Dialister (P* < 0.05) and *Roseburia (P* < 0.01) (p: Firmicutes) and *Anaeroplasma* (p: Tenericutes) (*P* < 0.01) were higher in the NRC diet group.

Consistent with the correlation between pregnancy stage and the gut microbiota in the proximal colon, *Corynebacterium* (p: Actinobacteria) (*P* < 0.05) and *Staphylococcus* (p: Firmicutes) (*P* < 0.01) were higher in the NRC vs. the CNF diet group. In the distal colonic luminal content samples, *Allobaculum (P* < 0.05) and *Staphylococcus (P* < 0.05) (p: Firmicutes) were higher in the NRC diet group. However, determination regarding [*Ruminococcus*] (p: Firmicutes) (*P* < 0.01) yielded opposing results. Consistent with the colonic position and gut microbiota correlation, *Bifidobacterium (P* < 0.05) and *Corynebacterium (P* < 0.01) (p: Actinobacteria), *Lactobacillus (P* < 0.01), *Oscillospira (P* < 0.01), and *Staphylococcus (P* < 0.05) (phylum Firmicutes), and *Desulfovibrio (P* < 0.05) (p: Proteobacteria) were lower in the proximal than in the distal colonic contents. However, *Bacteroides (P* < 0.01), *Paludibacter (P* < 0.05), and *[Prevotella] (P* < 0.01) (p: Bacteroidetes), *Coprococcus (P* < 0.05) and *Roseburia (P* < 0.05) (p: Firmicutes), and *Campylobacter (P* < 0.05) (phylum Proteobacteria) as well as *Anaeroplasma* (p: Tenericutes) (*P* < 0.05) exhibited the converse.

### Intestinal metabolites: SCFAs

Table 4 shows the SCFA concentrations in the colonic contents of pregnant Huanjiang mini-pigs fed two different nutrient level diets. In the proximal colon, isovalerate and total branched-chain fatty acids (BCFAs) decreased linearly from the first to the third trimester of pregnancy (*P* < 0.05). Acetate and total SCFAs trended toward decreasing with pregnancy trimester (0.05 ≤ *P* < 0.10). Acetate, propionate, and total SCFAs displayed significant differences according to diet × stage interaction (*P* < 0.05), with isobutyrate trending toward change (0.05 ≤ *P* < 0.10). In the distal colon, isobutyrate displayed significant differences according to diet (*P* < 0.05). Propionate and total BCFAs trended toward decreasing according to pregnancy stage (0.05 ≤ *P* < 0.10). Propionate, isobutyrate, total SCFAs, and total BCFAs displayed significant differences (*P* < 0.05) according to diet × stage interaction, whereas acetate, isopentanoate, and total BCFAs only trended toward change (0.05 ≤ *P* < 0.10).

### Bioamines

Table 5 summarises the bioamine concentrations in the colonic contents recovered from the pregnant Huanjiang mini-pigs fed two different nutrient level diets. In the mini-pig proximal colon, cadaverine, trytamine, tyramine, and total bioamines differed significantly (*P* < 0.05) according to diet. 1,7-heptyl diamine trended toward increasing according to pregnancy stage (0.05 ≤ *P* < 0.10). Although tyramine significantly differed (*P* < 0.05) according to diet × stage interaction, spermidine and spermine only trended towards change (0.05 ≤ *P* < 0.10). In the distal colon, eight bioamines and the total bioamines differed significantly according to the diet used (*P* < 0.05). Among bioamines, 1,7-heptyl diamine, trytamine, tyramine, and total bioamines displayed significant differences (*P* < 0.05) according to the pregnancy stage, whereas phenylethylamine and spermidine only trended towards changing with pregnancy trimester (0.05 ≤ *P* < 0.10). Lastly, 1,7-heptyl diamine, putrescine, spermidine, trytamine, tyramine, and total bioamines changed significantly according to diet × stage interactions (*P* < 0.05).

### Indole, skatole, and NH_3_-N

Table 6 summarises the indole, skatole, and NH_3_-N concentrations in the pregnant Huanjiang mini-pigs fed two different nutrient level diets. In the proximal colon, NH_3_-N differed significantly according to diet (*P* < 0.05), with indole and skatole only trending according to diet × stage interactions (*P* < 0.05). In the distal colon, indole significantly differed according to the diet used and pregnancy stage (*P* < 0.05). Lastly, NH_3_-N differed significantly according to diet × stage interaction (*P* < 0.05).

## Discussion

### Intestinal metabolites

Complex oligosaccharides and indigestible fibre matter not absorbed in the upper intestinal tract are fermented by the colonic anaerobic microbial community to produce SCFAs^18^; SCFAs can also be produced from some luminal amino acids^19^. Acetate, propionate, and butyrate are the major SCFAs produced in the colon. Among these, butyrate is thought to exhibit health benefits by acting as a major energy source for colonic epithelial cells, regulating gene expression in colonocytes, and exhibiting immunomodulatory and anti-inflammatory properties^20^. BCFAs including isobutyrate and isovalerate are produced during protein fermentation by gut bacteria^21^. Isobutyrate and isovalerate are produced from L-leucine and L-valine, respectively^22^. The amount of BCFAs in the faeces and intestinal contents thus constitutes an indicator of protein catabolism in the large intestine^19^. Decreased BCFA amounts suggest that fewer dietary and/or endogenous proteins escape digestion in the small intestine and enter into the large intestine. In this study, isovalerate and total BCFAs decreased linearly from the first to the third trimester of pregnancy in the proximal colon, and acetate and total SCFAs trended towards decrease. Additionally, propionate in the distal colon decreased linearly with pregnancy progression. Several studies have shown that the SCFA levels in the large intestine influence body weight gain and adiposity^23^,^24^. Specifically, obese mice demonstrated significantly increased caecal propionate and butyrate contents vs. their lean counterparts^23^. The reduced acetate levels would likely result in decreased lipogenesis and the increased propionate would contribute to inhibiting acetate conversion into lipid in the liver and adipose tissue^25^.

The colonic microbiota catabolises nitrogenous compounds to putrefactive catabolites such as ammonia, biogenic amines, indoles, and phenols^19^. NH_3_-N in the colonic lumen increases with an increase in dietary protein intake^26^ and is produced by the microbiota via urea degradation and amino acid deamination^19^. In the proximal colon, NH_3_-N was higher after NRC diet consumption than in the CNF diet groups; notably, excessive NH_3_-N in the colonic contents has been shown to inhibit colonocyte oxygen consumption^27^. *Leuconostoc, Bacillus, Atopostipes, Bacteroides*, and *Pseudomonas* metabolise tryptophan to indole-3-lactate and then to indole, or further to skatole in animal faeces^28^. In the distal colon, indole was significantly higher in both the NRC diet groups and according to pregnancy stage, consistent with the findings of Keseler *et al*., wherein lesser amounts of indoles were produced by the lower vs. the higher crude protein group^28^. Indole may be beneficial for the colonic epithelium by increasing epithelial cell tight-junction resistance^29^, whereas skatole has been associated with poor organoleptic characteristics of pig meat^30^, although its effects on the colonic mucosa remain unknown.

Bioamines comprise organic bases with one or more amine (NH_2_) group (s), and are formed mainly through the decarboxylation of free amino acids by bacterial decarboxylases^31^. Putrescine is synthesised from arginine or ornithine, which can occur simultaneously in many bacteria^32^. Putrescine appears to be strictly necessary for optimal undifferentiated colonic epithelial cell mitosis^33^. The precursor of cadaverine and tyramine is lysine^34^, and tyramine is synthesised by the decarboxylation of tyrosine^35^. Spermine is produced by spermidine, which in turn is produced from histidine, ornithine, arginine, and methionine^36^.

### The bacteria community

The bacterial community of the colon was determined from the first (day 45), second (day 75), and third (day 110) stages of pregnancy. To accurately capture the majority of bacterial OTUs within the colonic contents of Huanjiang mini-pigs, the study was normalised to samples at a depth of 31,465 sequences/sample with good coverage (>99.2%). We selected the V4 variable regions to interrogate the colon content bacterial communities. Alpha diversity analyses were performed using the proximal colonic contents samples across higher and lower nutrient diets and the three pregnancy trimesters. The OTUs decreased significantly according to pregnancy stage, and richness (Chao1 and ACE) changed significantly according to the pregnancy stage in both colonic segments analysed, suggesting that the microbiota diversity is lower at the end than at the beginning of pregnancy. This result is consistent with the results from Collado *et al*., who detected lower total faecal cell counts in the third than at the first pregnancy trimester^7^. In addition, Koren *et al*. also found that gut microbiota diversity and richness decreased with pregnancy^6^.

Furthermore, Le Chatelier *et al*. found that non-obese and obese Danish individuals differed in bacterial richness with low richness associated with higher overall levels of body fat^37^. The sows in our present study also gained considerable weight from the period of remating to late pregnancy. The unweighted UniFrac PCoA reflected the similarities of the bacterial communities within the colon. In contrast, we found that the CNF and NRC diet groups could almost be separated in the proximal colonic samples. However, unlike the samples recovered from the pregnant sows on days 45, 75, and 110 in the NRC diet group, the obtained proximal colonic samples in the CNF diet group could not be separated. Furthermore, our analyses identified no cluster separation in the distal colonic samples. These results demonstrate that the structure or diversity of the bacterial community likely changes to a greater extent in proximal colonic contents and that NRC diet consumption is associated with higher level of changes in the colonic content than is consumption of a CNF diet.

Firmicutes was prominent in the digesta-associated colon microbiota in all samples, for the two diets as well as throughout the three pregnancy trimesters. Firmicutes was composed mainly of the genera *Lactobacillus, Clostridium, Oscillospira, Ruminococcus, Turicibacter, Roseburia* (≥1%), and the other genera (<1%), which together comprised up to 45.43–74.57% of the total reads in some groups. The dominant taxa belonging to *Firmicutes* in the digesta-associated with the distal colon likely play an important role in starch and fibre degradation^38^. *Lactobacillus* abundance was higher in the proximal colon of pigs receiving the NRC diet vs. the CNF diet. OTU-31 (s: *Lactobacillus reuteri*) and OTU-1511 (s: *L. reuteri*) in the distal colon contents were decreased with pregnancy progression. *L. reuteri* exhibits strain-specific beneficial properties relevant to human health, with human associated lineages distinguished by genes related to bacteriophages, vitamin biosynthesis, antimicrobial production, and immunomodulation, although the strains show evidence of host adaptation^39^. In addition, *Lactobacillus* species have been associated with weight changes in humans and animals^40^. In comparison, the *Roseburia* component was larger in the whole colon of pigs receiving the NRC diet. Haro *et al*. reported that *Roseburia* was modulated by diet and that the degree of *Roseburia* increase was associated with a protective effect on the development of type 2 diabetes based on a human obese population with different diets^41^. In our study, the relative abundance of *Staphylococcus*, which accounted for 0.05–0.29% of the total sequences in the colon, linearly decreased from the first to the third trimester of pregnancy, which is not consistent with the results obtained in pregnant women^7^.

Bacteroidetes comprised the second dominant phylum in digesta-associated colonic microbiota. This phylum was more abundant in the proximal colonic content in CNF diet-fed animals, and more abundant in the proximal than the distal colon content samples. This phylum was prominent and mainly composed of the genera *Prevotella, YRC22*, and *Parabacteroides. Prevotella* comprised 1.83–7.28% of the total sequences in some groups. OTU-62, OTU-89, and OTU-1967 (all g: *Prevotella*) were linearly decreased with pregnancy. *Prevotella* has been proposed (together with other genera such as *Xylanibacter, Butyrivibrio*, and *Treponema*) to enhance caloric extraction from resistant starches and oligosaccharides, as well as from indigested carbohydrates^42^. Another study showed that long-term consumption of diets rich in carbohydrates can increase the abundance of *Prevotella*^43^. In addition, a study showed that *Prevotella* decreased in rats fed a high fat diet^44^.

Firmicutes and Bacteroidetes comprised the dominant colonic phyla, as previously suggested^45^. Earlier studies showed that obesity and glucose intolerance are associated with an increase in Firmicutes^37^. Obesity is also associated with a higher Firmicutes/Bacteroidetes ratio^46^. A high-fat diet can increase the Firmicutes/Bacteroidetes ratio within a small range^47^. Conversely, a higher saccharolytic diet may preferentially increase Bacteroidetes as compared to Firmicutes^48^. In our study, Firmicutes abundance was higher in distal colonic contents, whereas Bacteroidetes showed the opposite. Firmicutes was affected by diet and stage, with the NRC diet increasing its abundance in the proximal colon. In addition, Firmicutes was increased at the end of the pregnancy period in the distal colon. This increase was considered to alter the Firmicutes metabolic potential and enhance the body’s capacity to harvest energy from the diet^49^, a potential advantage during pregnancy to support growth of the foetus and prepare the body for the energetic demands of lactation. Jost *et al*. revealed that Firmicutes exhibited no detectable change over the perinatal period^8^. However, Khan *et al*. found that Firmicutes abundance was profoundly impacted during pregnancy^50^. Bacteroidetes was affected by diet only in the proximal colon, and the NRC diet decreased its abundance. The ratio of *Firmicutes*/*Bacteroidetes* was larger in groups fed the NRC vs. the CNF diet, suggesting that the gut microbiota of sows fed the NRC diet was more similar to obesity models than those fed the CNF diet.

Large abundances of other phyla were identified for Proteobacteria, Actinobacteria, Spirochaetes, and Tenericutes, which are present in the majority of gut-associated phylotypes in mammals^51^. These phyla are ubiquitous within the mammalian gut suggesting their critical role in the microbial ecology of the mammalian intestine^52^. Notably, *Enterobacteriaceae* and *E. coli* are higher in overweight than in normal-weight women and in women with excessive weight gain during pregnancy^53^. In the current study, *Escherichia* (phylum Proteobacteria) were more abundant in the proximal colon of pigs fed with HN NRC diet. As the NRC diet contains higher nutrient level than the CNF diet, the sows fed NRC demonstrated higher body weights. *E. coli* is sufficient to induce inflammation and glucose and insulin tolerance in germ free mice^54^, as well as acute diarrhoea in pregnant women^55^.

Several studies suggested that the gut microbiota change during pregnancy may also induce pregnancy complications such as excessive maternal weight gain^56^. Notably, our previous study showed that the average back-fat thickness of pregnant Huanjiang mini-pigs increased from days 45 (27.20 mm and 26.90 mm) to 75 (36.60 mm and 28.10 mm) post-service in both NRC and CNF diet groups, respectively^57^; the live body weight of these sows also increased from days 45 (73.82 kg and 67.52 kg) to 75 (86.14 kg and 75.28 kg) in both groups (Kong *et al*., unpublished data). These findings suggested that the pregnant sows became obese during pregnancy, especially in the NRC group, and that this was associated with more abundant *Escherichia*. Actinobacteria was more abundant in the distal colonic contents recovered from animals fed the CNF diet and was in addition more abundant in the distal than in the proximal colon. Among bacteria, *Bifidobacterium* and *Corynebacterium* were more abundant in the distal colon from the CNF vs. the NRC groups, consistent with a previous study showing that high-fat feeding is associated with lower *Bifidobacteria* concentrations^9^. Notably, the *Bifidobacterium* group is present in higher numbers in normal-weight than in overweight women^7^, and in women with lower weight gain over pregnancy^7^. The present study indicates that consumption of the diet with higher nutrient level coincides with the presence of a colonic microbiota in pregnant sows that resembles the gut bacteria composition observed in obese animals.

## Conclusion

Consumption of a higher nutrient level diet increases the colonic luminal content of bacterial metabolites related to nitrogen metabolism, suggesting increased dietary and endogenous protein transfer from the small to the large intestine^58^ and/or modified composition/metabolic capacities of the intestinal microbiota toward luminal protein/amino acids. The dominate phyla in the colonic luminal contents were, to different extents, Firmicutes, Bacteroidetes, Proteobacteria, Actinobacteria, Spirochaetes, and Tenericutes. The dominate genera in the colonic content of pregnant sows were *Lactobacillus, Treponema, Ruminococcus, Clostridium*, and *Prevotella*. The richness of bacteria in the colon decreased from the first to the third trimester of pregnancy. Lastly, consumption of an NRC diet resulted in a colonic microbiota profile of sows resembling the gut bacteria composition observed in human obesity. This might be associated with an increased risk of pregnancy syndrome, although no causal link between these parameters was established in our study, calling for further experimental works.

## Materials and Methods

### Animals, housing, and treatment

The present study was carried out in accordance with the Chinese guidelines for animal welfare and experimental protocols and approved by the Animal Care and Use Committee of the Institute of Subtropical Agriculture, Chinese Academy of Sciences.

Forty-eight primiparous Huanjiang mini-pigs with a mean body weight (BW) of 43.02 ± 8.87 kg were obtained from a mini-pig farm located in Jixiang town (108°27′40.8″ E and 25°9′50″ N, altitude 578 m), Huanjiang county (Guangxi province, China). These sows were randomly assigned to one of the two dietary groups post service (24 sows per each dietary group and three sows per pen). One group of sows received diet with a higher nutrient level and the other received diet with a lower nutrient level. The higher nutrient level diet was formulated to meet the nutrient requirements recommended by the National Research Council (1998) (NRC diet) and contained 14.50 MJ/kg digestible energy, 13.10% crude protein, and 4.56% crude fibre. The lower nutrient level diet was formulated to meet recommendations of the Chinese National Feeding Standard for Swine (CNF diet), and thus contained 12.20 MJ/kg of digestible energy, 11.00% of crude protein, and 6.86% of crude fibre (Supplementary Table S3). These diets are widely used in commercial crossbred pig farms and commercial Huanjiang mini-pig farms, respectively.

All animals were housed in 2 m × 3 m pens with cement-sclerified flooring. Each pen was equipped with a feeder and a nipple drinker. The room temperature was maintained at 22–28 °C. All pigs had *ad libitum* access to drinking water and were fed twice daily (08:30 and 16:30) with their diets (about 2.5% of body weight) after service. The feed intake, excreta, and mental state per sow were monitored twice each day throughout the entire experimental period.

### Sample collection and preparation

According to the report of Johnston and Trottier (1999)^59^, the early, middle, and later stages of pregnancy in pigs are from days 1 to 30, from days 30 to 75, and from days 75 to delivery, respectively. Considering that the Huanjiang mini-pigs represent miniature animals and it is difficult to collect the conceptus samples to determine foetus development for subsequent study, we chose 45 days post-service to sample as the early pregnancy stage.

This study was conducted using samples collected from 31 sows offered two experimental diets. On day 45, 75 or 110 of service, the pregnant sows from each treatment group were sacrificed for sample collection at 12 h after the last feeding^60^. Briefly, the pigs were administered general anaesthesia by intravenous injection of 4% sodium pentobarbital solution (40 mg/kg BW), and sacrificed by exsanguination via the carotid artery^61^. After colon recovery, the luminal contents were collected from a region 10 cm posterior to the ileocecal valve and a region 10 cm at the end of the colon, respectively, and then stored at −80 °C. Subsequently, the contents of ammonia (considered as the sum of NH_3_ and NH_4_^+^), SCFAs, indole, skatole (3-methylindole), and bioamines were analysed, and the composition of the gut microbiota was determined.

### DNA Extraction and PCR Amplification

Total genomic DNA was extracted from the colon content samples using a QIAamp DNA Stool Mini Kit (Qiagen, Hilden, Germany), according to the manufacturer’s instructions. The quality and quantity of DNA were measured using a NanoDrop ND-1000 spectrophotometer (NanoDrop Technologies Inc., Wilmington, DE, USA). An absorption ratio (260/280 nm) within 1.8–2.0 was deemed to be of sufficient purity to be used for subsequent analyses. The total DNA of the colon contents was then diluted to 50 ng/μl and used in the preparation of amplicons for high-throughput sequencing of microbial 16S rRNA. Conventional PCR was performed to amplify the V4 regions of the 16S rRNA gene using universal primers 515F (5′-GTG CCA GCM GCC GCG GTA A-3′) and 806R (5′-GGA CTA CHV GGG TWT CTA AT-3′). The reverse primer contained a 6-bp error-correcting barcode unique to each sample. DNA was amplified using the protocol described previously^62^. DNA samples were sent to a commercial service provider (Novogene, Beijing, China) for amplicon pyrosequencing on an Illumina MiSeq platform according to the manufacturer’s instruction^63^.

### Microbial community analysis

Raw data were screened and assembled by QIIME^64^ and FLASH^63^ software packages. The reads flagged as chimeras were removed to form the “effective sequences” collection for each sample. QIIME software package (http://qiime.org/) and UPARSE pipeline (http://drive5.com/uparse) were used for analysis of the sequences and determination of the Operational Taxonomic Units (OTUs) with an identity threshold of 97%. Meanwhile, we picked a representative sequence for each OTU and used the RDP classifier^65^ to assign taxonomic data to each representative sequence. Taxonomy classifications were assigned against RDP Classifier (Version 2.2, http://sourceforge.net/projects/rdpclassifier/)^65^ and the Greengenes database (http://greengenes.lbl.gov/cgi-bin/nph-index.cgi)^66^. Alpha diversity values for colon bacterial communities of pregnant Huanjiang mini-pigs were obtained using abundance-based coverage estimator (ACE), bias-corrected Chao richness estimator, Shannon index, Simpson index, and Good’s coverage, at a sequence depth of 31,460 for all samples. Principal coordinate analysis (PCoA) of microbial communities (jackknifed beta diversity from resampling 100 times at depth of 31,460 sequences) was performed using Bray-Curtis distance.

### Metabolites in colon contents

SCFAs including acetate, propionate, butyrate, isobutyrate, valerate, and isovalerate were analysed as described previously^67^. To ensure the homogenicity of the intestine content sample, the freeze-dried samples were prepared using a Vacuum freeze-dryer (Hrist ALPHA 2-4/LSC, Germany) at −80 °C. Briefly, freeze-dried samples (0.5–0.6 g) were weighed into 10 ml centrifuge tubes and mixed with 8 ml ddH_2_O, homogenised, and centrifuged in sealed tube at 7,000 *g* and 4 °C for 10 min. A mixture of the supernatant fluid and 25% metaphosphoric acid solution (0.9 and 0.1 ml, respectively) was centrifuged at 20,000 *g* and 4 °C for 10 min after standing in a 2 ml sealed tube at 4 °C for over 2 h. The supernatant portion was then filtered through a 0.45-μm polysulfone filter and analysed using Agilent 6890 gas chromatography (Agilent Technologies, Inc, Palo Alto, CA, USA) with a flame ionisation detector and a 1.82 m × 0.2 mm I.D. glass column that was packed with 10% SP-1200/1% H_3_PO_4_ on the 80/100 Chromosorb W AW (HP, Inc., Boise, ID, USA). The concentration of NH_3_-N in the supernatant fluid was measured at 550 nm using a UV-2450 spectrophotometer (Shimadzu, Kyoto, Japan)^68^. The bioamines including 1,7-heptyl diamine, cadaverine, phenylethylamine, putrescine, trytamine, tyramine, spermidine, and spermine, as well as the indoles and skatoles, were analysed as described previously^69^.

### Statistical analysis

The alpha diversity indices of bacterial communities, the apparent relative abundances at phylum and genus levels, as well as colon metabolites (SCFA, NH_3_-N, indole, skatole, and bioamines) were analysed in a completely randomised design with the GLM procedure of SAS (SAS Institute, Inc., Cary, NC). The PCoA of overall diversity of microbial communities based on an un-weighted Unifrac metric was performed using Bray-Curtis distance to compare all samples. A study of the relationship among parameters was also carried out using Pearson’s linear correlation coefficient. Phylum and genus at <0.5% relative abundance for both diets were excluded from all analyses. Differences with *P* values <0.05 were considered as statistically significant while a tendency was considered to exist at 0.05 ≤ *P* < 0.10.

## Additional Information

**How to cite this article**: Kong, X.-f. *et al*. Colonic luminal microbiota and bacterial metabolite composition in pregnant Huanjiang mini-pigs: effects of food composition at different times of pregnancy. *Sci. Rep.* **6**, 37224; doi: 10.1038/srep37224 (2016).

**Publisher's note:** Springer Nature remains neutral with regard to jurisdictional claims in published maps and institutional affiliations.

## Supplementary Material

Supplementary Information

## Figures and Tables

**Table 1:** Alpha diversity indices of colon bacterial communities in Huanjiang mini-pigs at different stages of pregnancy.

Items	NRC diet			CNF diet			SEM	*P* values		
	45 d	75 d	110 d	45 d	75 d	110 d		Diet	Stage	D*S
Proximal colon										
Row tags	41039.2	41118.7	42682.7	41276.6	42296.3	40799.0	268.06	0.787	0.602	0.140
Effective Tags	39920.6	39569.7	41271.3	40541.8	41110.1	39401.7	281.97	0.872	0.983	0.110
Number of OTUs	1209.8	1002.0	1024.3	1145.9	1161.3	1036.7	21.84	0.377	0.017	0.040
Chao1	1342.5	1118.6	1146.7	1305.2	1304.0	1142.2	24.66	0.285	0.009	0.055
ACE	1356.7	1326.6	1165.1	1317.2	1326.6	1165.1	13.59	0.257	0.008	0.037
Simpson	0.98	0.95	0.94	0.92	0.93	0.92	0.01	0.088	0.642	0.556
Shannon	7.42	6.56	6.52	6.81	6.75	6.70	0.13	0.768	0.223	0.342
Coverage	0.993	0.994	0.993	0.993	0.993	0.994	<0.01	0.132	0.010	0.073
Distal colon										
Row tags	42545.2	41068.3	41185.0	41299.1	41226.0	40558.7	289.32	0.368	0.352	0.567
Effective Tags	41552.6	39506.7	39747.7	40266.6	39892.1	38992.7	296.38	0.384	0.103	0.442
Number of OTUs	1224.6	1101.7	1101.7	1183.4	1221.3	1101.3	16.53	0.419	0.062	0.063
Chao1	1372.5	1235.8	1248.0	1332.3	1360.7	1217.4	17.95	0.601	0.038	0.062
ACE	1392.0	1252.8	1255.3	1348.9	1388.1	1237.4	18.19	0.469	0.031	0.044
Simpson	0.98	0.98	0.96	0.97	0.97	0.97	<0.01	0.634	0.490	0.632
Shannon	7.52	7.23	6.86	7.29	7.37	7.34	0.10	0.553	0.593	0.470
Coverage	0.992	0.993	0.993	0.993	0.992	0.994	<0.01	0.922	0.066	0.076

**Table 2:** Effects of diet and pregnancy stage on the OTU fractions of the proximal colon bacterial communities in Huanjiang mini-pigs (%).

Items	NRC diet			CNF diet			SEM	*P* values			Annotation	Phylum
	45 d	75 d	110 d	45 d	75 d	110 d		Diet	Stage	D*S		
OTU-2	5.31	5.71	4.36	1.50	2.30	1.02	0.58	0.004	0.662	0.981	g: *Lactobacillus*	Firmicutes
OTU-9	3.78	4.17	1.50	1.93	1.28	1.27	0.37	0.028	0.270	0.365	f: *Lachnospiraceae*	Firmicutes
OTU-11	1.65	0.14	0.22	0.30	0.79	0.38	0.18	0.616	0.268	0.037	g: *Bifidobacterium*	Actinobacteria
OTU-16	0.51	0.03	0.07	0.11	0.30	1.00	0.09	0.128	0.261	0.017	g: *Sutterella*	Proteobacteria
OTU-17	1.08	0.60	0.36	0.61	0.53	0.48	0.06	0.237	0.017	0.123	f: *Lachnospiraceae*	Firmicutes
OTU-20	0.68	0.34	0.31	0.44	0.38	0.47	0.04	0.816	0.028	0.061	g: *Oscillospira*	Firmicutes
OTU-25	0.88	0.22	0.36	0.72	0.60	0.73	0.07	0.129	0.019	0.100	f: *Ruminococcaceae*	Firmicutes
OTU-27	0.43	0.35	0.33	0.52	0.46	0.72	0.04	0.013	0.426	0.291	f: *Ruminococcaceae*	Firmicutes
OTU-28	0.16	0.02	0.13	0.12	0.01	0.49	0.04	0.229	0.043	0.194	f: *Streptococcaceae*	Firmicutes
OTU-29	0.57	0.10	0.08	0.06	0.01	0.16	0.05	0.089	0.051	0.042	s: *Megasphaera elsdenii*	Firmicutes
OTU-33	0.88	0.36	0.40	0.62	0.53	0.62	0.06	0.671	0.045	0.131	g: *Ruminococcus*	Firmicutes
OTU-36	0.40	0.77	1.65	0.28	0.34	0.39	0.09	<0.01	0.001	0.003	g: *Treponema*	Spirochaetes
OTU-38	0.39	0.25	0.22	0.43	0.47	0.60	0.05	0.043	0.877	0.430	o: *Clostridiales*	Firmicutes
OTU-41	0.78	2.80	1.18	0.89	0.65	1.10	0.19	0.028	0.030	0.003	g: *Roseburia*	Firmicutes
OTU-44	0.29	0.49	0.21	0.13	0.17	0.50	0.04	0.330	0.175	0.009	f: *Lachnospiraceae*	Firmicutes
OTU-45	0.13	0.25	0.32	0.04	0.05	0.17	0.03	0.005	0.052	0.588	s: *Lactobacillus delbrueckii*	Firmicutes
OTU-46	0.14	0.31	0.44	0.39	0.39	1.72	0.12	0.015	0.015	0.092	g: *YRC22*	Bacteroidetes
OTU-47	0.44	0.13	0.13	0.15	0.10	0.23	0.03	0.110	0.003	0.005	f: *Lachnospiraceae*	Firmicutes
OTU-55	0.20	0.52	1.04	0.41	0.71	0.82	0.09	0.739	0.041	0.631	g: *Prevotella*	Bacteroidetes
OTU-59	0.12	0.38	0.21	0.13	0.09	0.07	0.03	0.006	0.093	0.021	g: *Treponema*	Spirochaetes
OTU-62	0.57	0.42	0.15	0.27	0.33	0.25	0.06	0.122	0.033	0.053	g: *Prevotella*	Bacteroidetes
OTU-65	0.57	0.17	0.20	0.23	0.25	0.14	0.03	0.065	0.002	0.003	g: *Ruminococcus*	Firmicutes
OTU-68	0.14	0.09	0.05	0.13	0.35	0.13	0.02	0.003	0.007	0.002	f: *Lachnospiraceae*	Firmicutes
OTU-79	0.25	0.32	0.11	0.17	0.13	0.11	0.02	0.048	0.125	0.251	g: *Anaerovibrio*	Firmicutes
OTU-82	0.21	0.31	0.48	0.44	0.49	0.52	0.04	0.046	0.173	0.604	g: *Parabacteroides*	Bacteroidetes
OTU-85	0.29	0.05	0.07	0.08	0.09	0.13	0.02	0.430	0.024	0.007	f: *Lachnospiraceae*	Firmicutes
OTU-98	0.09	0.04	0.06	0.15	0.17	0.28	0.02	0.010	0.597	0.529	g: *Oscillospira*	Firmicutes
OTU-105	0.12	0.04	0.26	0.28	0.49	0.31	0.05	0.028	0.721	0.197	o: *Bacteroidales*	Bacteroidetes
OTU-106	0.12	0.02	0.07	0.22	0.13	0.25	0.03	0.019	0.216	0.867	g: *Fibrobacter*	Fibrobacteres
OTU-195	0.08	0.06	0.12	0.28	0.25	0.12	0.03	0.048	0.761	0.437	g:*[Prevotella]*	Bacteroidetes
OTU-216	0.29	0.02	0.11	0.12	0.08	0.10	0.02	0.324	0.008	0.050	f: *S24-7*	Bacteroidetes
OTU-805	0.18	0.17	0.65	0.30	0.18	0.19	0.04	0.205	0.104	0.042	g: *Treponema*	Spirochaetes
OTU-1149	0.06	0.22	0.30	0.25	0.34	0.42	0.03	0.027	0.033	0.842	p: *Bacteroidetes*	Bacteroidetes
OTU-1366	0.34	0.05	0.04	0.16	0.13	0.09	0.03	0.772	0.009	0.074	f: *Christensenellaceae*	Firmicutes
OTU-1890	0.21	0.71	0.34	0.22	0.13	0.25	0.06	0.050	0.196	0.037	g: *Roseburia*	Firmicutes
OTU-2187	7.03	13.59	20.99	24.35	20.47	23.47	1.92	0.024	0.407	0.247	f: *Sphingomonadaceae*	Proteobacteria
OTU-3683	0.21	0.21	0.35	0.26	0.23	0.38	0.02	0.320	0.026	0.946	f: *Ruminococcaceae*	Firmicutes

**Table 3:** Effects of diet and pregnancy stage on the OTU fractions of the distal colon bacterial communities in Huanjiang mini-pigs (%).

Items	NRC diet			CNF diet			SEM	*P* values			Annotation	Phylum
	45 d	75 d	110 d	45 d	75 d	110 d		Diet	Stage	D*S		
OTU-9	2.59	4.11	6.40	4.18	1.50	1.53	0.48	0.039	0.638	0.026	f: *Lachnospiraceae*	p: Firmicutes
OTU-11	0.62	1.81	0.66	0.20	3.11	3.12	0.46	0.030	0.840	0.683	g: *Bifidobacterium*	p: Actinobacteria
OTU-14	0.21	0.37	1.10	0.37	0.27	0.23	0.07	0.046	0.076	0.016	s: *Clostridium butyricum*	p: Firmicutes
OTU-25	0.69	1.34	0.70	0.45	1.17	1.59	0.12	0. 005	0.532	0.924	f: *Ruminococcaceae*	p: Firmicutes
OTU-27	0.44	0.55	0.33	0.34	0.30	0.70	0.04	0.054	0.040	0.121	f: *Ruminococcaceae*	p: Firmicutes
OTU-28	0.07	0.61	0.24	0.03	0.02	1.96	0.18	0.043	0.083	0.190	f: *Streptococcaceae*	p: Firmicutes
OTU-29	0.19	0.09	0.21	0.06	0.03	0.30	0.03	0.785	0.024	0.481	s: *Megasphaera elsdenii*	p: Firmicutes
OTU-31	0.30	0.63	0.29	1.74	0.48	0.73	0.11	0.298	0.001	<0.01	s: *Lactobacillus reuteri*	p: Firmicutes
OTU-34	0.48	0.31	0.25	0.86	0.32	0.19	0.07	0.047	0.077	0.234	g: *Prevotella*	p: Bacteroidetes
OTU-35	0.28	0.29	0.05	0.09	0.16	0.15	0.03	0.313	0.022	0.847	f: *[Paraprevotellaceae]*	p: Bacteroidetes
OTU-36	0.68	0.27	1.28	1.48	0.28	0.34	0.15	0.005	0.368	0.402	g: *Treponema*	p: Spirochaetes
OTU-38	0.34	1.04	0.44	0.33	0.74	1.41	0.13	0.013	0.509	0.685	o: *Clostridiales*	p: Firmicutes
OTU-41	0.87	0.42	0.68	1.68	0.34	0.76	0.13	0.028	0.354	0.073	g: *Roseburia*	p: Firmicutes
OTU-48	0.33	0.56	0.62	0.27	0.37	0.87	0.06	0.115	0.033	0.828	f: *Christensenellaceae*	p: Firmicutes
OTU-49	0.00	0.00	0.95	0.20	0.05	0.08	0.09	0.049	0.075	0.144	g: *Veillonella*	p: Firmicutes
OTU-62	1.03	0.56	0.23	0.38	0.32	0.14	0.10	0.315	0.049	0.597	g: *Prevotella*	p: Bacteroidetes
OTU-72	0.17	0.49	0.14	0.12	0.26	0.44	0.05	0.012	0.358	0.619	f: *Ruminococcaceae*	p: Firmicutes
OTU-74	0.36	0.11	0.24	0.13	0.17	0.31	0.03	0.413	0.147	0.019	g: *Bacteroides*	p: Bacteroidetes
OTU-75	0.35	0.13	0.17	0.28	0.13	0.17	0.02	0.008	0.451	0.135	f: *Lachnospiraceae*	p: Firmicutes
OTU-77	0.08	0.50	0.08	0.06	0.32	0.48	0.06	0.003	0.639	0.756	s: *Ruminococcus flavefaciens*	p: Firmicutes
OTU-79	0.22	0.16	0.16	0.40	0.10	0.14	0.02	0.004	0.138	0.014	g: *Anaerovibrio*	p: Firmicutes
OTU-85	0.16	0.27	0.10	0.08	0.43	0.29	0.04	0.024	0.861	0.420	f: *Lachnospiraceae*	p: Firmicutes
OTU-89	0.61	0.23	0.10	0.07	0.15	0.08	0.06	0.332	0.020	0.138	g: *Prevotella*	p: Bacteroidetes
OTU-111	0.28	0.07	0.09	0.03	0.24	0.15	0.03	0.732	0.758	0.011	g: *Oscillospira*	p: Firmicutes
OTU-113	0.03	0.27	0.05	0.04	0.38	0.23	0.05	0.007	0.766	0.755	f: *Ruminococcaceae*	p: Firmicutes
OTU-120	0.10	0.40	0.10	0.01	0.29	0.32	0.05	0.009	0.615	0.947	c: *Clostridia*	p: Firmicutes
OTU-133	0.28	0.09	0.09	0.21	0.02	0.01	0.03	0.025	0.277	0.746	g: *Oscillospira*	p: Firmicutes
OTU-146	0.28	0.33	0.08	0.05	0.15	0.18	0.03	0.198	0.010	0.882	g: *Staphylococcus*	p: Firmicutes
OTU-164	0.15	0.26	0.20	0.10	0.34	0.24	0.03	0.012	0.929	0.229	c: *Clostridia*	p: Firmicutes
OTU-216	0.34	0.04	0.07	0.03	0.09	0.24	0.03	0.724	0.141	0.011	f: *S24-7*	p: Bacteroidetes
OTU-659	0.27	0.08	0.07	0.39	0.14	0.05	0.04	0.034	0.068	0.454	g: *Oscillospira*	p: Firmicutes
OTU-1350	0.42	0.37	0.33	0.52	0.38	0.68	0.03	0.437	0.446	0.033	g: *Ruminococcus*	p: Firmicutes
OTU-1366	0.25	0.46	0.09	0.09	0.19	0.21	0.04	0.065	0.020	0.746	f: *Christensenellaceae*	p: Firmicutes
OTU-1386	0.23	0.34	0.13	0.13	0.16	0.15	0.03	0.315	0.047	0.779	f: *Ruminococcaceae*	p: Firmicutes
OTU-1428	0.26	0.26	0.27	0.22	0.25	0.62	0.03	0.009	0.004	0.015	f: *Lachnospiraceae*	p: Firmicutes
OTU-1454	0.14	0.20	0.26	0.21	0.16	0.38	0.02	0.285	0.018	0.189	g: *Ruminococcus*	p: Firmicutes
OTU-1511	0.12	0.32	0.13	0.72	0.19	0.35	0.05	0.671	0.024	<0.01	s: *Lactobacillus reuteri*	p: Firmicutes
OTU-1890	0.19	0.09	0.15	0.27	0.07	0.11	0.02	0.009	0.703	0.262	g: *Roseburia*	p: Firmicutes
OTU-1967	0.47	0.14	0.06	0.25	0.13	0.11	0.03	0.016	0.009	0.026	g: *Prevotella*	p: Bacteroidetes
OTU-2133	0.17	0.25	0.18	0.16	0.20	0.36	0.02	0.039	0.328	0.541	o: *Clostridiales*	p: Firmicutes
OTU-2531	0.53	0.15	0.35	0.24	0.13	0.06	0.05	0.011	0.304	0.428	f: *Ruminococcaceae*	p: Firmicutes
OTU-2590	1.23	1.65	0.33	0.53	0.33	0.26	0.17	0.876	0.009	0.638	g: *Treponema*	p: Spirochaetes
OTU-2768	0.16	0.21	0.21	0.14	0.24	0.48	0.03	0.005	0.036	0.231	o: *Clostridiales*	p: Firmicutes
OTU-3219	0.23	0.68	0.31	0.14	0.42	1.04	0.08	0.003	0.141	0.520	f: *Christensenellaceae*	p: Firmicutes
OTU-3683	0.21	0.21	0.41	0.30	0.22	0.46	0.03	0.829	0.015	0.636	f: *Ruminococcaceae*	p: Firmicutes

**Table 4:** Concentrations of short-chain fatty acids in the colonic contents of Huanjiang mini-pigs at different stages of pregnancy (mg/g).

Items	NRC diet			CNF diet			SEM	*P* values		
	45 d	75 d	110 d	45 d	75 d	110 d		Diet	Stage	D*S
Proximal colon										
Acetate	7.32	7.58	6.96	9.51	6.94	6.60	0.30	0.484	0.055	0.047
Propionate	2.48	2.92	2.58	3.14	2.32	1.86	0.13	0.400	0.226	0.035
Butyrate	0.19	0.15	0.15	0.18	0.15	0.13	0.01	0.501	0.115	0.892
Isobutyrate	1.22	1.53	1.19	1.40	0.99	0.92	0.07	0.173	0.439	0.073
Valerate	0.18	0.16	0.15	0.17	0.15	0.15	0.01	0.849	0.509	0.983
Isovalerate	0.29	0.26	0.19	0.25	0.20	0.20	0.01	0.157	0.013	0.532
A/P	2.99	2.63	2.71	3.04	3.14	3.53	0.08	0.003	0.399	0.102
BCFAs	0.37	0.31	0.30	0.35	0.30	0.28	0.01	0.635	0.202	0.986
SCFAs	11.31	12.29	10.91	14.30	10.46	9.58	0.48	0.951	0.098	0.037
BCFAs/SCFAs	0.03	0.03	0.03	0.02	0.03	0.03	0.01	0.922	0.861	0.146
Total short-chain fat acids	11.68	12.60	11.22	14.65	10.76	9.87	0.49	0.938	0.091	0.040
Distal colon										
Acetate	5.63	6.11	5.61	6.78	5.18	4.92	0.21	0.722	0.227	0.062
Propionate	2.07	2.69	2.05	2.52	1.98	1.64	0.09	0.187	0.075	0.006
Butyrate	0.20	0.52	0.21	0.28	0.24	0.22	0.05	0.567	0.302	0.224
Isobutyrate	0.96	1.35	1.15	1.13	0.79	0.82	0.05	0.019	0.781	0.003
Valerate	0.19	0.27	0.17	0.26	0.25	0.23	0.02	0.264	0.335	0.334
Isovalerate	0.22	0.29	0.22	0.26	0.22	0.21	0.01	0.545	0.362	0.072
A/P	2.67	2.35	2.74	2.74	2.62	3.04	0.08	0.210	0.155	0.790
BCFA	0.39	0.79	0.37	0.55	0.49	0.45	0.05	0.825	0.117	0.077
SCFA	8.88	10.44	9.02	10.70	8.18	7.59	0.33	0.326	0.224	0.011
BCFA/SCFA	0.05	0.08	0.04	0.05	0.06	0.06	0.01	0.992	0.213	0.443
Total short-chain fat acids	9.27	11.23	9.39	11.24	8.67	8.04	0.34	0.294	0.174	0.004

A/P: acetate/propionate; BCFA: branched-chain fatty acids, including isobutyrate and isovalerate; SCFA: straight-chain fatty acids, including acetate, propionate, butyrate, and valerate.

**Table 5:** Concentrations of bioamines in the colonic contents of Huanjiang mini-pigs at different stages of pregnancy (μg/g).

Items	NRC diet			CNF diet			SEM	*P* values		
	45 d	75 d	110 d	45 d	75 d	110 d		Diet	Stage	D*S
Proximal colon										
1,7-heptyl diamine	0.75	0.72	1.29	1.01	1.01	1.13	0.06	0.275	0.080	0.337
Cadaverine	4.07	2.56	2.06	1.05	1.51	1.31	0.29	0.009	0.461	0.167
Phenylethylamine	3.58	3.07	2.82	2.55	2.25	1.69	0.25	0.101	0.565	0.973
Putrescine	6.73	9.06	8.68	6.75	7.38	8.23	0.50	0.550	0.375	0.759
Spermidine	12.96	20.47	21.60	17.22	15.82	17.33	0.93	0.448	0.194	0.087
Spermine	3.57	7.49	5.21	4.84	4.25	3.85	0.45	0.258	0.218	0.082
Trytamine	4.49	4.36	5.63	1.18	3.54	1.50	0.47	0.007	0.505	0.288
Tyramine	2.72	5.00	7.77	3.41	1.73	1.95	0.51	0.007	0.355	0.036
Total bioamine	38.87	52.74	55.07	40.26	37.35	37.00	2.42	0.029	0.317	0.343
Distal colon										
1,7-heptyl diamine	0.98	0.81	2.56	1.41	0.80	1.03	0.10	0.023	<0.01	<0.01
Cadaverine	2.35	2.11	1.23	1.02	0.97	1.45	0.18	0.046	0.769	0.265
Spermidine	18.12	24.19	29.61	17.17	12.17	18.10	1.22	<0.01	0.077	0.025
Spermine	5.85	6.39	9.56	4.37	2.99	5.08	0.49	0.003	0.117	0.417
Phenylethyl amine	1.43	2.48	2.25	1.35	1.38	1.68	0.13	0.028	0.080	0.137
Putrescine	3.63	6.60	7.89	4.40	3.51	4.12	0.41	0.015	0.152	0.031
Trytamine	1.21	2.25	7.84	1.41	0.68	0.33	0.39	<0.01	0.002	<0.01
Tyramine	1.59	4.01	8.71	2.09	1.44	1.05	0.44	<0.01	0.009	<0.01
Total bioamine	35.17	48.83	69.64	33.22	23.95	32.83	2.63	<0.01	0.005	0.002

**Table 6:** Concentrations of indole, skatole, and NH_3_-N in the colonic contents of Huanjiang mini-pigs at different stages of pregnancy.

Items	NRC diet			CNF diet			SEM	*P* values		
	45 d	75 d	110 d	45 d	75 d	110 d		Diet	Stage	D*S
Proximal colon										
NH_3_-N (mg/g)	20.74	18.86	16.36	13.49	15.54	11.85	1.06	0.005	0.475	0.814
Indole (μg/g)	9.21	15.42	7.67	11.85	9.53	14.88	0.89	0.436	0.557	0.011
Skatole (μg/g)	3.89	3.97	2.75	2.83	2.73	5.43	0.27	0.806	0.473	0.014
Distal colon										
NH_3_-N (mg/g)	11.91	18.38	16.80	15.40	13.61	11.49	0.77	0.156	0.296	0.021
Indole (μg/g)	7.27	11.20	9.55	9.91	11.19	14.27	0.54	0.016	0.017	0.146
Skatole (μg/g)	4.98	4.42	3.89	3.88	4.95	4.34	0.26	0.951	0.743	0.382
