# Supplementary material for: Colonic luminal microbiota and bacterial metabolite composition in pregnant Huanjiang mini-pigs: effects of food composition at different times of pregnancy
Source: Sci Rep. 2016 Dec 5;6:37224. doi: 10.1038/srep37224 (PMC5137017; doi:10.1038/srep37224)
Supplement: Supplementary Information [file srep37224-s1.doc]

**Supplementary Information**

**Colonic luminal microbiota and bacterial metabolite composition in pregnant Huanjiang mini-pigs: effects of food composition at different times of pregnancy**

Xiang-feng Kong^1,2,#,*^, Yu-jiao Ji^1,#^, Hua-wei Li^1^, Qian Zhu^1^, F. Blachier^3^, Mei-mei Geng^1^, Wen Chen^1^ & Yu-long Yin^1,2*^

**Table S1. Bacterial phyla correlated by Pearson’s correlation to diet, pregnancy stage, and position in colonic contents**.

| Phyla | Diet | | Stage | | Position |
| --- | --- | --- | --- | --- | --- |
|  | PCLC | DCLC | PCLC | DCLC | PCLC/DCLC |
| *Actinobacteria* | -0.120 | 0.425* | -0.113 | 0.075 | 0.276* |
| *Bacteroidetes* | 0.404* | -0.086 | 0.117 | -0.097 | -0.439** |
| *Elusimicrobia* | -0.174 | -0.384* | -0.078 | -0.178 | 0.031 |
| *Firmicutes* | -0.365* | -0.055 | -0.017 | 0.350* | 0.337** |
| *Lentisphaerae* | 0.135 | 0.001 | 0.045 | -0.098 | 0.274* |
| *Tenericutes* | 0.230 | 0.274 | -0.495** | -0.451* | 0.096 |
| *Verrucomicrobia* | 0.024 | -0.201 | -0.354 * | -0.051 | 0.274** |

PCLC, proximal colonic luminal contents; DCLC, distal colonic luminal contents.

**P*<0.05; ***P*<0.01.

**Table S2. Bacterial genera correlated by Pearson’s correlation to diet, pregnancy stage, and position in colonic contents**.

| Phyla | Genera | Diet | | Stage | | Position |
| --- | --- | --- | --- | --- | --- | --- |
|  |  | PCLC | DCLC | PCLC | DCLC | PCLC/DCLC |
| Actinobacteria | *Bifidobacterium* | -0.098 | 0.407* | -0.201 | 0.086 | 0.260* |
|  | *Corynebacterium* | -0.197 | 0.360 * | -0.366* | -0.031 | 0.374** |
| Bacteroidetes | *Bacteroides* | 0.278 | -0.189 | 0.189 | 0.006 | -0.405** |
|  | *Paludibacter* | 0.443* | 0.235 | 0.008 | 0.267 | -0.311* |
|  | *Parabacteroides* | 0.428* | -0.074 | 0.263 | 0.162 | 0.046 |
|  | *[Prevotella]* | 0.230 | -0.122 | 0.198 | -0.010 | -0.427** |
| Firmicutes | *Allobaculum* | -0.292 | -0.126 | -0.303 | -0.354* | -0.049 |
|  | *Coprococcus* | 0.084 | 0.012 | -0.132 | 0.042 | -0.311* |
|  | *Dialister* | -0.278 | -0.395* | -0.272 | -0.166 | -0.045 |
|  | *Facklamia* | 0.200 | 0.333 | -0.294 | -0.261 | 0.272* |
|  | *Lactobacillus* | -0.569** | -0.281 | 0.172 | 0.346 | 0.326** |
|  | *Oscillospira* | 0.062 | 0.017 | -0.260 | -0.130 | 0.507** |
|  | *Roseburia* | -0.425* | -0.495** | 0.174 | 0.097 | -0.266* |
|  | *Staphylococcus* | -0.123 | 0.258 | -0.492** | -0.441* | 0.296* |
|  | *Veillonella* | -0.297 | -0.273 | 0.254 | 0.360* | -0.117 |
|  | *[Ruminococcus]* | -0.192 | -0.135 | 0.278 | 0.531** | -0.020 |
| Proteobacteria | *Campylobacter* | 0.145 | -0.232 | 0.120 | 0.014 | -0.300* |
|  | *Desulfovibrio* | -0.188 | -0.102 | -0.103 | 0.073 | 0.303* |
|  | *Escherichia* | -0.349* | 0.137 | -0.139 | 0.045 | 0.120 |
| Tenericutes | *Anaeroplasma* | -0.428* | -0.530** | -0.203 | -0.026 | -0.250* |

PCLC, proximal colonic luminal contents; DCLC, distal colonic luminal contents.

**P*<0.05; ***P*<0.01.

**Table S3. Composition and nutritional ingredients of the two experimental diets (air dried basis, %)**.

| Ingredients^1^ | HN | LN | Nutrient level^4^ | HD | | LD |
| --- | --- | --- | --- | --- | --- | --- |
| Corn | 58.20 | 57.20 | Digestible energy (MJ/Kg) | 14.50 | | 12.20 |
| Soybean meal | 11.00 | 0.00 | Crude protein | 13.10 | | 11.00 |
| Wheat bran | 11.50 | 11.00 | Crude protein/Digestible energy | 0.90 | | 0.90 |
| Rice bran | 4.00 | 13.00 | Crude fiber | 4.56 | | 6.86 |
| Alfalfa meal^2^ | 3.00 | 14.00 | Ether extract | 9.34 | | 5.00 |
| Lys | 0.88 | 0.88 | Ca | 0.62 | | 0.58 |
| Met | 0.27 | 0.27 | Tatol P | 0.52 | | 0.44 |
| Thr | 0.33 | 0.33 | Available P | 0.28 | | 0.26 |
| Try | 0.08 | 0.08 | Lys | 1.11 | | 0.83 |
| Soybean oil | 7.50 | 0.00 | Met+Cys | 0.65 | | 0.52 |
| Dicalcium phosphate | 1.15 | 1.15 |  | | |  |
| Limestone | 0.79 | 0.79 |  | | |
| Salt | 0.30 | 0.30 |  | | |
| Premix^3^ | 1.00 | 1.00 |  |  |  |

HN: high nutrient level diet; LN: low nutrient level diet.

^1^All dietary components except of the alfalfa meal were provided by Guilin Dragon Biotechnology Co., Ltd. (Guilin city, Guangxi province, China), and all components were mashed and pelletized.

^2^Alfalfa meal was purchased from Gansu Tianmu Co., Ltd.(Gansu province, China).

^3^The premix provides the following per kg of diet: VA 12 040 IU, VD_3_ 2 112 IU, VE 29.7 IU, VK_3_ 2.8 mg, VB_1_ 1.2 mg, VB_2_ 7.1 mg, VB_6_ 1.3 mg, VB_12_ 0.03 mg, nicotinic acid 42.9 mg, pantothenic acid 21.6 mg, folic acid 0.44 mg, biotin 0.12 mg, choline 320 mg, Fe 80 mg, Cu 40 mg, Zn 140 mg, Mn 52 mg, I 0.56 mg, Co 1.4 mg, Se 0.33 mg, Ca 8 mg, P 0.8 mg.

^4^Digestible energy, crude protein, Ca, total phosphorus, and available phosphorus are calculated values, whereas the others are measured.

**Figure Legend**

**Figure S1.** **Principal coordinate analysis of the UniFrac distance metric.**

Graphs were generated using OTUs from each sample.

(**a**) Luminal content samples of the proximal and (**b**) distal colon. NRC45, NRC75, and NRC110: data from samples obtained from Huanjiang mini-pigs fed with NRC diet for 45, 75, and 110 days, respectively. CNF45, CNF75, and CNF110: data from samples obtained from Huanjiang mini-pigs fed with CNF diet for 45, 75, and 110 days, respectively.

**(a)**
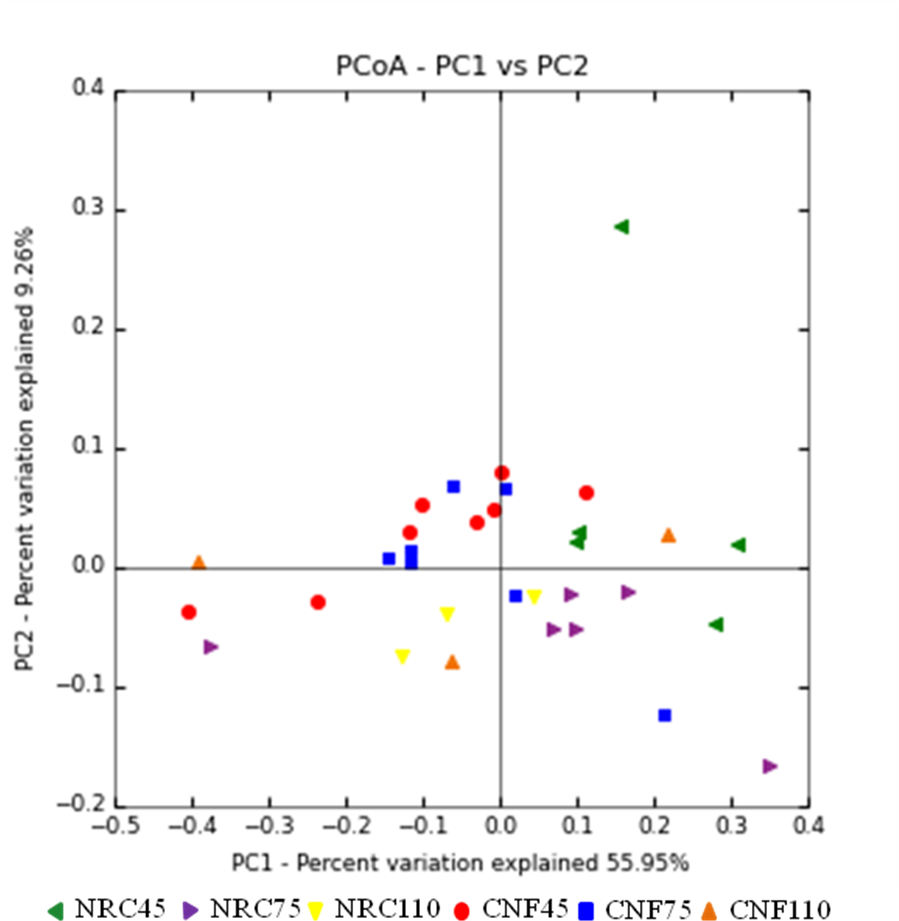
**(b)**
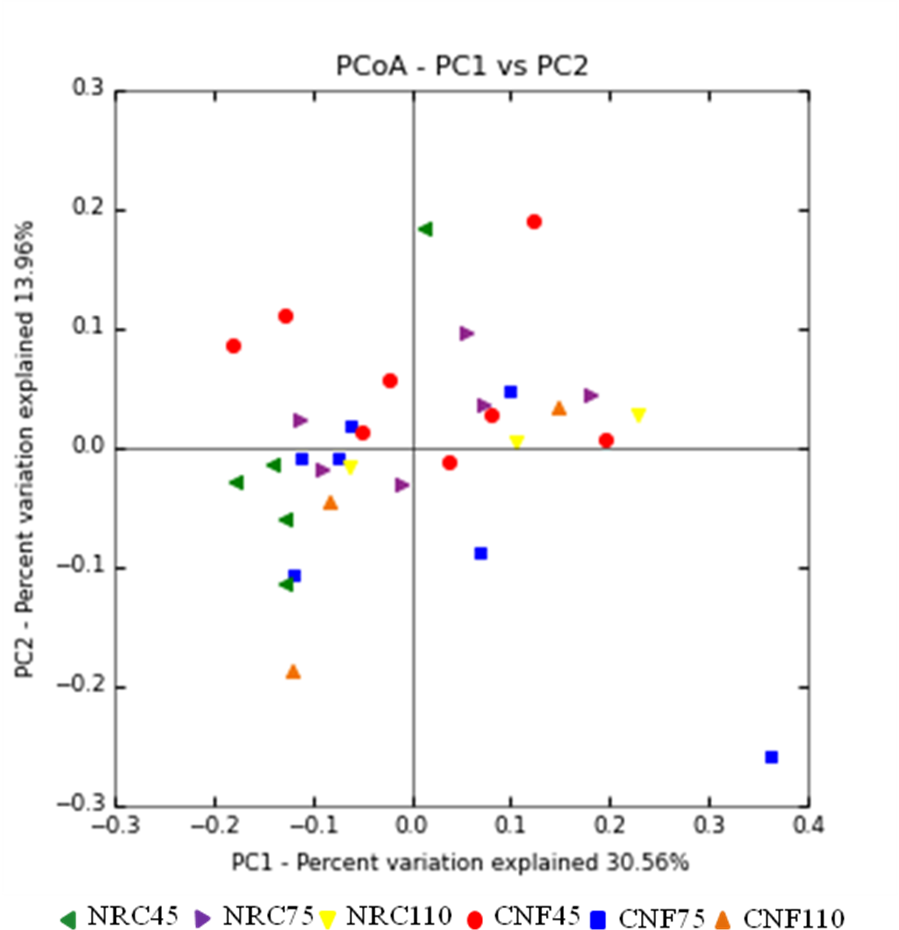


**Fig. S1**
